# Soluble Fiber with High Water-Binding Capacity, Swelling Capacity, and Fermentability Reduces Food Intake by Promoting Satiety Rather Than Satiation in Rats

**DOI:** 10.3390/nu8100615

**Published:** 2016-10-02

**Authors:** Chengquan Tan, Hongkui Wei, Xichen Zhao, Chuanhui Xu, Yuanfei Zhou, Jian Peng

**Affiliations:** 1 Department of Animal Nutrition and Feed Science, College of Animal Science and Technology, Huazhong Agricultural University, Wuhan 430070, China; cooper2005@163.com (C.T.); weihongkui@mail.hzau.edu.cn (H.W.); FenziYY@126.com (X.Z.); xuchuanhui001@hzau.edu.cn (C.X.); 2 The Cooperative Innovation Center for Sustainable Pig Production, Wuhan 430070, China

**Keywords:** guar gum, water binding capacity, meal pattern, food intake, satiety

## Abstract

To understand whether soluble fiber (SF) with high water-binding capacity (WBC), swelling capacity (SC) and fermentability reduces food intake and whether it does so by promoting satiety or satiation or both, we investigated the effects of different SFs with these properties on the food intake in rats. Thirty-two male Sprague-Dawley rats were randomized to four equal groups and fed the control diet or diet containing 2% *konjac* flour (KF), pregelatinized waxy maize starch (PWMS) plus guar gum (PG), and PWMS starch plus xanthan gum (PX) for three weeks, with the measured values of SF, WBC, and SC in the four diets following the order of PG > KF > PX > control. Food intake, body weight, meal pattern, behavioral satiety sequence, and short-chain fatty acids (SCFAs) in cecal content were evaluated. KF and PG groups reduced the food intake, mainly due to the decreased feeding behavior and increased satiety, as indicated by decreased meal numbers and increased inter-meal intervals. Additionally, KF and PG groups increased concentrations of acetate acid, propionate acid, and SCFAs in the cecal contents. Our results indicate that SF with high WBC, SC, and fermentability reduces food intake—probably by promoting a feeling of satiety in rats to decrease their feeding behavior.

## 1. Introduction

Satiation and satiety are part of the body’s appetite control system involved in limiting food intake. Satiation is reflected in meal size and meal duration, while satiety is reflected in meal number and inter-meal intervals [1]. Modulation of food intake by consuming foods with a high satiety or satiation value may be one of the approaches to help reduce obesity. The efficacy by which different types of dietary fibers promote satiety or satiation is varied [2,3]. This issue can be explored first by using laboratory animals due to the advantages of a complete control over the diet, facile collection of gut samples, and systematic observation of behaviors.

Growing evidence showed that the physicochemical properties of dietary fiber such as viscosity, water-binding capacity, and fermentability may contribute to decrease the food intake in human beings and rodents [3,4,5]. Viscous fibers increase chewing activity and saliva production in the mouth [6], which could result in early satiation and reduced food intake [7,8]. However, dietary fiber with high water-binding capacity may increase gastric distension by expanding their volume up to eight-fold in the stomach [9], which will increase feelings of satiety [10], probably via afferent vagal signals of fullness [6]. Moreover, a delay in gastric emptying accompanied by an increase in gastric distension is often associated with enhanced satiety between meals [11]. In addition, dietary fiber is also fermented by intestinal microbiota, contributing to the increase of short-chain fatty acids (SCFAs). Furthermore, SCFAs could stimulate the release of satiety-related peptides, such as peptide tyrosine-tyrosine (PYY) and glucagon-like peptide-1 (GLP-1) from entero-endocrine cells, which may promote feelings of satiety [12].

*Konjac* flour (KF) is a water soluble, viscous dietary fiber with a high water-binding capacity (15.5 mL H_2_O/g) [13,14]. KF mainly contains *konjac* glucomannan (KGM), which is composed of β-1,4-linked d-mannosyl and d-glucosyl residues at a molar ratio of 1.6:1.0 as the main chain with a small number of branches through β-1,3 mannosyl units [15]. KGM is considered a functional fiber of food ingredient and has been consumed in the form of rubbery jelly, noodles, and other food products by humans in Asia for centuries [16]. KGM has been known to be helpful in lowering cholesterol levels, reducing weight in human beings [17], and modifying intestinal microbial metabolism in sows [18,19]. Our previous study indicated that dietary inclusion of KF with higher water-binding capacity (1.97 vs. 1.78 g/g) and swelling capacity (2.14 vs. 1.62 mL/g) than inclusion of wheat bran promoted the satiety of gestating sow [20]. However, the tuber is usually grown in Asian countries and the resources for commercial production are limited, suggesting the necessity to develop novel dietary fibers with functional properties similar to those of KF, as well as similar effects on food intake reduction by promoting satiety or satiation.

Pregelatinized waxy maize starch, a fermentable resistant starch composed mainly of highly branched amorphous amylopectin, has many specific food attributes [21]. Dietary supplementation with modified waxy maize starch lowers glucose-dependent insulinotropic polypeptides and has beneficial implications in weight management [22]. Guar and xanthan gums with high viscosity and hydrating properties are used as stabilizer and thickener in various food products [23,24]. A previous study reported that the digesta viscosity of subjects was increased after consuming cookies containing 2.5% guar gum alone for 42 days, without any effect on their food intake [3]. The consumption of beverages enriched with dietary fibers and xanthan gum (0.22%) for one week also had no influence on the appetite sensations in healthy men [25]. Our previous results showed that the combined fiber materials (pregelatinized waxy maize starch plus guar gum (PG) and pregelatinized waxy maize starch plus xanthan gum (PX)) had higher or similar water-binding capacity or swelling capacity than the KF [26]. Therefore, we hypothesized that the combination of pregelatinized waxy maize starch with guar gum or xanthan gum inclusion in diet may have a similar effect to that of KF on physicochemical properties (water-binding capacity and swelling capacity) and fermentability, as well as food intake reduction by promoting satiety or satiation. The objective of the present study was to investigate whether dietary fibers with high water-binding capacity, swelling capacity, and fermentability reduce food intake by promoting satiety or satiation as well as the mechanism underlying it by evaluating their effects on plasma concentrations of GLP-1 and PYY, SCFAs in cecal contents, meal pattern, feeding behavior, food intake, and body weight in rats.

## 2. Experimental Section

### 2.1. Animals and Diets

A total of 32 male Sprague-Dawley rats (average initial body weight of 377.10 ± 3.43 g; 10 weeks of age) (Hunan SJA Laboratory Animal Co., Ltd., Changsha, China) were housed individually in standard cages that allowed recording of food intake. The cages were placed in a temperature-and humidity-controlled room (21 ± 2 °C, 55% ± 10% humidity) with a 12:12 light/dark cycle (lights off at 8:30 p.m.). The animals were acclimatized for one week with the control diet fed prior to the experiment. Rats were given free access to water and food. All the experiment protocols were approved by the animal care and use committee of Huazhong Agriculture University and were in accordance with the National Research Council’s Guide for the Care and Use of Laboratory Animals. Sprague-Dawley rats were used because they are reported to have a good consistency in meal patterns [27].

Rats were divided randomly to four dietary treatment groups (8 rats/treatment): the control fed control diet with wheat bran as a fiber source (Control); a positive control fed the control diet with 2% *konjac* flour (Qingjiang Konjac Products Co., Ltd., Wuhan, China) to replace 2% wheat bran (KF); a treatment fed the control diet with 2% pregelatinized waxy maize starch (Hangzhou Puluoxiang Starch Corp., Ltd., Hangzhou, China) plus guar gum (Shangdong Yunzhou Science and Technology Corp., Ltd., Yunzhou, China) to replace 2% wheat bran (PG) (PG; 85.7% pregelatinized waxy maize starch and 14.3% guar gum); and a treatment fed the control diet with 2% pregelatinized waxy maize starch plus xanthan gum (Shangdong Yunzhou Science and Technology Corp., Ltd., Yunzhou, China) to replace 2% wheat bran (PX) (PX; 95% pregelatinized waxy maize starch and 5% xanthan gum). The ingredient and chemical composition of the diets are listed in Table 1. The experiment lasted 21 days, during which food intake was determined daily and body weights were measured.

### 2.2. Behavioral Satiety Sequence

The analysis of the behavioral satiety sequence was performed on day 15 of dietary treatment essentially as described by Halford et al. [28]. Rats were housed individually in transparent observational cages and fasted overnight for 12 h. Water was available ad libitum. The following morning (8:30–9:30), rats were given a pre-weighed amount of food and their behaviors were monitored. For all rats, behavior was recorded every 30 s for 1 h as reported previously [29] and food intake was calculated. Feeding and non-feeding behaviors were continuously scored using a video system connected with a computer in a nearby room by a highly trained experimenter blind to the dietary treatment of the animals. Behaviors were categorized as: feeding (animal at hopper trying to obtain food, chewing, gnawing, or holding food in paws), drinking (animal licking spout of water bottle), grooming (animal scratching, licking, or biting any part of its anatomy), activity (including locomotion, sniffing, or rearing), inactivity (immobility when aware, or signs of sickness behavior), resting (animal curled up, resting head with eyes closed). Data were collated into 5-min period bins for display. With time spent in each of the behaviors as % of the total behavior.

### 2.3. Meal Pattern Analysis

On day 17 of dietary treatment, the rats were housed individually in transparent observational cages that allowed continuous recording of food intake for three days. The first two days served as the adaptation period, and the meal pattern and food intake were analyzed on the third day. The measurements were performed similar to previous studies [30]. Rats were fasted overnight for 12 h before the meal pattern analysis. Meal patterns of the nocturnal (from 8:30 p.m. to 8:30 a.m.) and diurnal (from 8:30 a.m. to 8:30 p.m.) periods were analyzed and recorded. Rats were fed respective diets ad libitum during the meal pattern analysis. The results were collected as total food intake (g), feeding rate (mg/s), meal size (g), meal duration (s), meal number, and inter-meal interval (min). A meal was defined as an intake larger than 0.3 g lasting a period longer than 13 s, and two distinct meals needed to be separated by >10 min [27]. The meal patterns for rats were recorded using monitoring equipment in a computer-based data acquisition system (Shenzhen Quick Zoom Technology Co., Ltd., N5063 960P, Shenzhen, China).

### 2.4. Samples Collection

On day 20 of dietary treatment, rats were fasted overnight and then given food ad libitum the following morning. Two hours after the meal presentation, the rats were anesthetized (ethylene oxide) and opened by laparotomy. Blood samples were collected by cardiac puncture and placed into tubes containing ethylene diamine tetraacetic acid (EDTA) and a peptidase inhibitor cocktail containing general protease inhibitor (Roche Diagnostics Ltd., Burgess Hill, West Sussex, UK), followed by centrifugation at 3000× *g* for 10 min at 4 °C and storage at −80 °C until GLP-1 and PYY analysis. The gut was removed to sample the contents from cecum and tissue from distal ileum. Cecal contents were stored at −80 °C until analysis of short chain fatty acids (SCFAs). Tissues from distal ileum were immersed in RNAlater (QIAGEN, Crawley, UK) for 5 days at 4 °C and then stored at −80 °C until analysis.

### 2.5. Chemical Analysis

Diet samples were analyzed for crude protein according to the Association of Analytical Communities (AOAC) [31]. Gross energy was determined by bomb calorimetry using a LECO Ac 300 automated calorimeter system 789-500 (Parr Instrument Co., Moline, IL, USA). Soluble dietary fiber and insoluble fiber were determined by AOAC Method 991.43 [31]. Viscosity in extracts of diets was measured as reported by Johansen et al. [32]. The water-binding capacity and swelling capacity of diets were measured as described by Serena et al. [33].

The concentrations of GLP-1 and PYY in plasma were analyzed using an ultrasensitive rat GLP-1 and PYY enzyme-linked immunosorbent assay (ELISA) kit (Biosource Inc., Sunnyvale, CA, USA) according to the instructions. The SCFAs concentration of cecal contents was analyzed by gas chromatography (Varian CP-3800, Shimadzu Co., Kyoto, Japan), as described by Bosch et al. [34]. Total SCFAs was determined as the sum of analyzed acetate, propionate, and butyrate. All procedures were performed in duplicate.

### 2.6. Quantitative PCR (QPCR)

QPCR technology was employed to verify changes in the mRNA levels of GLP-1 and PYY genes. Total RNA was isolated from the distal ileum tissue using TRIZOL reagent (Invitrogen, Life Technologies Co., Carlsbad, CA, USA) according to the manufacturer’s recommendations. Briefly, 2.5 μg of RNA was reverse transcribed using a First-strand cDNA synethesis kit (TOYOBO, Kyoto, Japan) and cDNAs were stored at −20 °C. After 10-fold dilution, cDNA was used for the relative quantification of gene amplification. Primer sets for all genes were designed using Primer 6.0 Software (Applied Biosystems, Foster City, CA, USA) and synthesized commercially by Sangon (Shanghai, China). The primers sequences for rat PYY were sense 5′ CCGTTATGGTCGCAATGCT 3′, antisense 5′ TCTCGCTGTCGTCTGTGAA 3′. The primers sequences for rat GLP-1 were: sense 5′ CGGAAGAAGTCGCCATAGC 3′, antisense 5′ CAGCCAGTTGATGAAGTCTCT 3’. Rat β-*actin* was selected as the internal control gene for all QPCR reactions by using the following primers sequences: 5′ CTTTCTACAATGAGCTGCGTGTG 3′, antisense 5′ GTCAGGATCTTCATGAGGTAGTCTGTC 3′. cDNA was amplified by QPCR using a Bio-Rad CFX Connect™ Real-Time PCR Detection System (Bio-Rad, Richmond, CA, USA) under the following conditions: 95 °C for 3 min for enzyme activation, followed by denaturing at 95 °C for 20 s and annealing at 59 °C for 20 s and elongation at 72 °C for 20 s, repeated for a total of 40 cycles. Gene expression levels were calculated after normalization to the standard housekeeping gene β-*actin* using the ΔΔ*C*_t_ method. Briefly, the mean values of the triplicate cycle thresholds (CT) of the target genes (PYY and GLP-1) were normalized to the mean values of triplicate CT of the reference β-*actin* using the calculation formula “2^CT^_β-*actin*_^−CT^_target gene_”, which indicated a relative value as a fraction of PYY or GLP-1.

### 2.7. Statistical Analysis

Data were compared by repeated ANOVA measures plus Tukey’s post-hoc test, using the “PROC MIXED” function of the SAS software program (SAS 9.1, SAS Institute Inc., Cary, NC, USA). Data were examined to ensure patterns of constant variance and a normal distribution. The results that did not meet these conditions were transformed to appropriate data using logarithms or square roots. Data were presented as means ± SEM, and significant differences were accepted at a probability of *p* < 0.05.

## 3. Results

### 3.1. Food Intake and Body Weight

**Figure 1:**
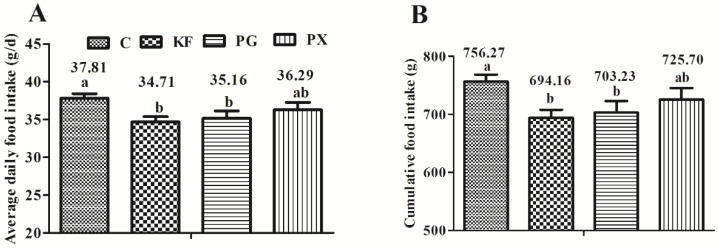

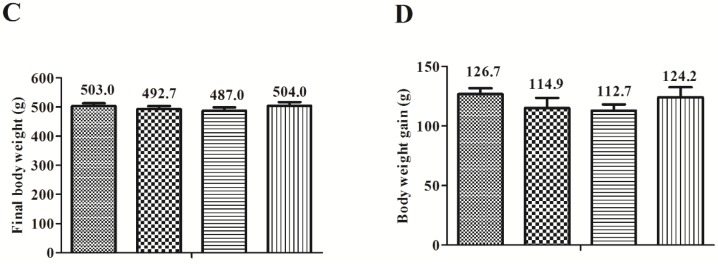
Food intake and body weight. (**A**) Average daily food intake; (**B**) cumulative food intake; (**C**) final body weight; and (**D**) body weight gain in rats given diets containing different dietary fibers for three weeks. Diets were control (C) or supplemented with 2% fiber of *konjac* flour (KF), pregelatinized waxy maize starch plus guar gum (PG) or pregelatinized waxy maize starch plus xanthan gum (PX); Values are mean ± SEM of *n* = 8 per group. Bars with different letters indicate a significant difference (*p* < 0.05).

Average daily food intake throughout the experiment was affected by the dietary treatment in the decreasing magnitude order of control > PX > PG > KF (*p* < 0.05). Accordingly, cumulative food intake was significantly lower for rats fed the KF and PG diets than their control counterparts (*p* < 0.05), but with no significant difference between control and PX groups (Figure 1B). Compared with the control group, the final body weight and body weight gain showed a lower tendency in the KF and PG groups, but was not statistically significant (*p* > 0.05) (Figure 1C,D).

### 3.2. Behavioral Satiety Sequence

**Figure 2:**
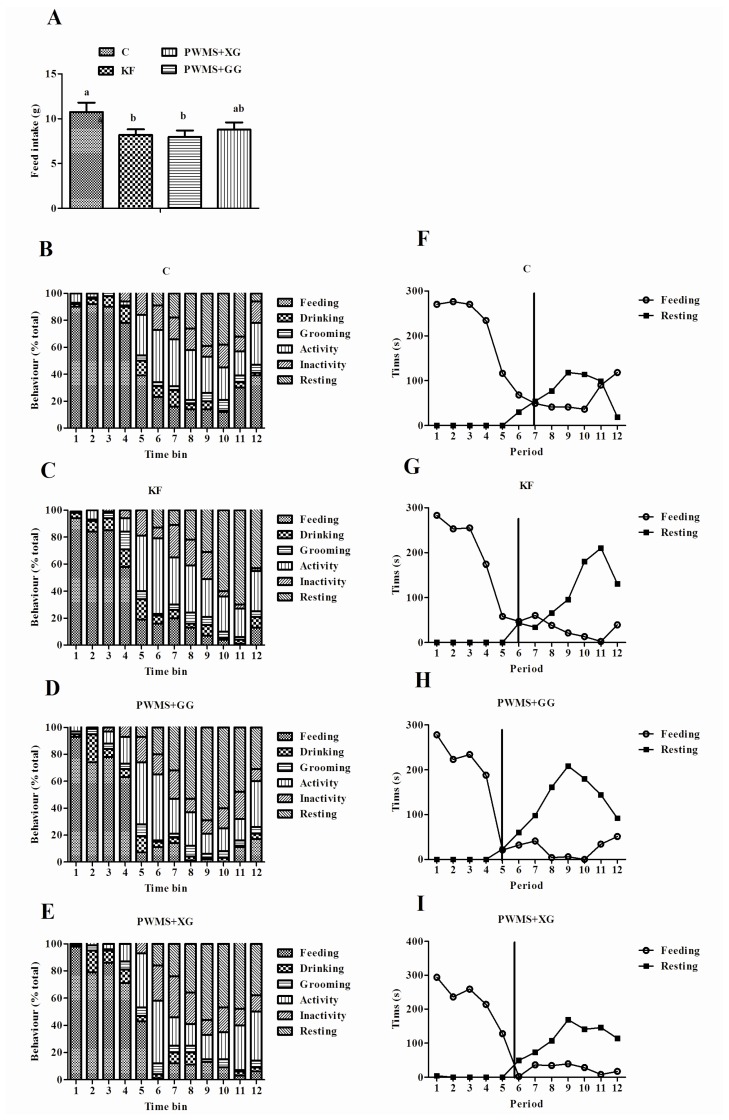
Food intake (**A**) in rats was calculated at 1 h and is presented as mean ± SEM for *n* = 8 per group. Bars with different letters indicate a significant difference (*p* < 0.05). Periods 1–12 correspond to the twelve 5-min time bins comprising the 60 min test session; (**B**–**E**) Frequency data within each behavioral category are expressed as a proportion of the total number of observations per time bin; (**F**–**I**) correspond to crossover graphs illustrating the point of transition from eating to resting. The perpendicular line indicates satiety point. Diets were control (C) or supplemented with 2% fiber of *konjac* flour (KF), pregelatinized waxy maize starch plus guar gum (PG), or pregelatinized waxy maize starch plus xanthan gum (PX).

The behavioral satiety sequence (feeding, drinking, activity, inactivity, grooming, and resting) of rats is shown in Table 2 and Figure 2. The overall pattern of behaviors was similar among all the dietary treatments. Quantification of individual behaviors indicated that the KF and PG groups spent significantly less time on feeding than the control group (*p* < 0.05; Table 2). When compared with other groups, the control group showed the feeding peak in the second time bin (control 91.88%, KF 84.38%, PG 74.38%, and PX 78.75%, respectively, *p* = 0.07; Figure 2). Furthermore, compared with bin 7 in the control group (Figure 2F–I), the KF, PG, and PX groups showed the transition from eating to resting in time bins 6, 5, and 6, respectively, which was consistent with the result that KF and PG groups had a lower food intake than the control group during the 1 h observation period (*p* < 0.05; Figure 2A).

### 3.3. Meal Pattern Parameters

Meal pattern parameters are presented in Table 3. Although the diurnal and nocturnal food intakes were similar among the four groups, the PG, KF, and PX groups were significantly lower than the control group in total food intake (*p* < 0.01). The feeding rate, meal size and meal duration (total, diurnal, and nocturnal) were similar among all the groups. However, the total and nocturnal meal numbers of the KF and PG groups were significantly lower than those of the control group (*p* < 0.05). As expected, the PG group was obviously higher than the control group in total inter-meal interval (*p* < 0.05), and the KF and PX groups were also higher, but not statistically significant.

### 3.4. Plasma Concentrations of GLP-1/PYY, mRNA Abundance in Distal Ileum of GLP-1/PYY, and Concentrations of SCFAs in Cecal Contents

**Figure 3:**
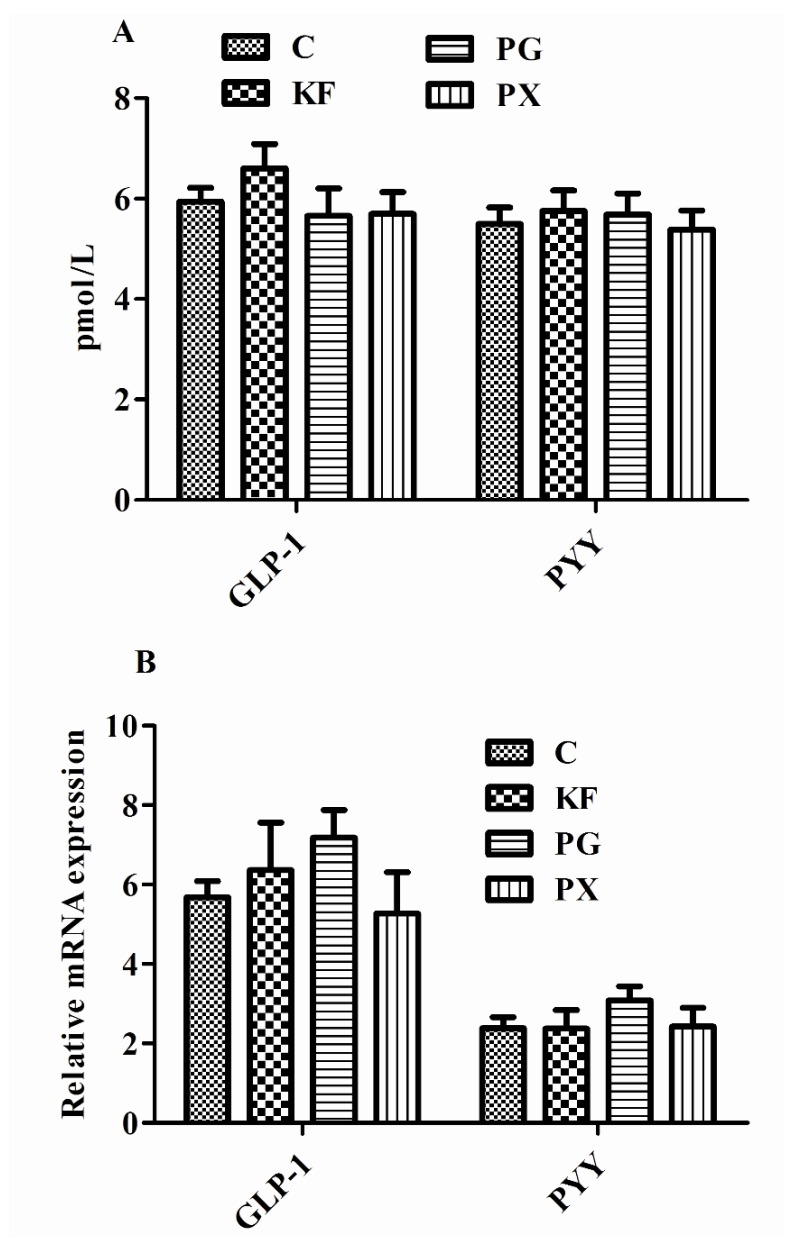
Plasma concentrations (**A**) and mRNA abundance in distal ileum (**B**) of GLP-1 and PYY in rats given diets containing different dietary fibers for three weeks; Diets were control (C) or supplemented with 2% fiber of *konjac* flour (KF), pregelatinized waxy maize starch plus guar gum (PG), or pregelatinized waxy maize starch plus xanthan gum (PX); Values are mean ± SEM, *n* = 6–8 per group. GLP-1, glucagon-like peptide-1; PYY, peptide tyrosine-tyrosine.

**Figure 4:**
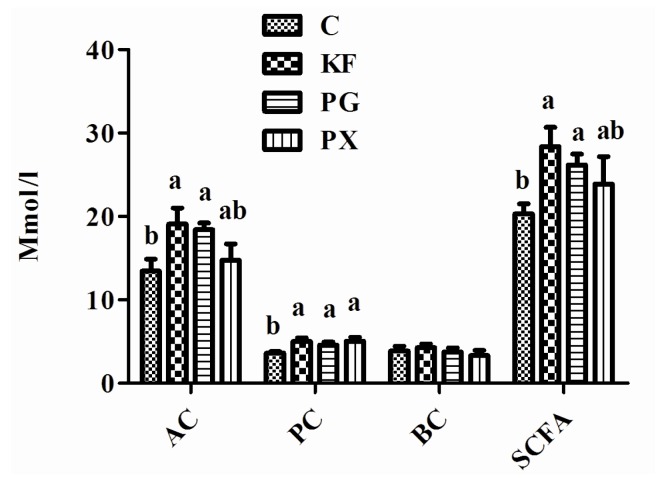
Concentration of SCFAs in cecal contents of rats given diets containing different dietary fibers for three weeks. Diets were control (C) or supplemented with 2% fiber of *konjac* flour (KF), pregelatinized waxy maize starch plus guar gum (PG) or pregelatinized waxy maize starch plus xanthan gum (PX); Values are mean ± SEM of *n* = 6–8 per group. Bars with different letters indicate a significant difference (*p* < 0.05). SCFAs, short-chain fatty acids; AC, acetate acid; PC, propionate acid; BC, butyrate acid.

There was no significant difference in the plasma concentrations of GLP-1 and PYY, and the mRNA abundance of GLP-1 and PYY in ileum tissue among the four dietary treatments (Figure 3). Compared with the control group, the concentrations of acetic acid and SCFAs in cecal contents were increased in the KF and PG groups (*p* < 0.05; Figure 4). Additionally, the KF, PG, and PX groups had higher concentrations of the propionic acid in cecal contents than the control group (*p* < 0.05).

## 4. Discussion

The present study aimed to explore whether dietary fibers regulate appetite by promoting satiety or satiation, and to this end, behavioral satiety sequence and meal pattern were monitored. Detailed analysis of the behavioral satiety sequence revealed that the overall pattern of the behavioral response to fast-induced food intake in rats was normal. However, we observed a decrease in short-term (1 h) food intake, accompanied by reduced feeding behavior in rats fed the KF and PG diets. Furthermore, the transition from eating to resting occurred in early time bins in the PG, PX, and KF groups, indicating that satiety was induced in the three groups [35]. To our knowledge, this is probably the first report so far about a detailed analysis of the behavioral satiety sequence following dietary fiber treatments in rats.

The present results are consistent with our previous study in that supplementation of KF reduced the non-feeding oral behavior and promoted the satiety of sows [20]. In the current study, there was no significant difference in the feeding rates of rats among all the dietary treatments. Based on the association of taste and flavor aversions with a decrease in feeding rate [36], the consistent feeding rates indicated that dietary fiber supplementation did not produce a significant effect on food palatability, implying that the changes in the meal pattern observed here might be attributed to physiological factors rather than changes in diet palatability or flavor.

In the present study, dietary supplementation of KF and PG reduced the total food intake, mainly due to the increased satiety, as indicated by decreased meal numbers and increased inter-meal intervals. This is in agreement with a previous study that reported the suppression of food intake 2 h after the administration of the mixture of guar gum and fructo-oligosaccharide, due to decreased meal numbers and increased inter-meal intervals [37]. It is worth noting that the current result contrasted with a most recent study about diets supplemented with guar gum or fructo-oligosaccharide alone [30]. Although both of them reduced the food intake immediately after dietary fiber ingestion, the result was mainly attributed to a decrease of meal size and meal duration but not meal number and inter-meal intervals, indicating that the decrease in food intake is associated with increased satiation rather than satiety. In the current study, the rats consumed such a high proportion of their daily intake during the light phase, probably due to their 12-h fast overnight before the meal pattern analysis.

Pregelatinized waxy maize starch and fructo-oligosaccharide are highly fermentable fibers [38,39], and guar gum is a viscous fiber with high water-binding capacity [3]. This suggests that the combination of fibers with different physicochemical properties may affect the physiological processes (satiation or satiety) of appetite regulation. In the present study, dietary treatments were similar in insoluble fiber and viscosity, but their soluble fiber, water-binding capacity, and swelling capacity followed the sequence of PG > KF > PX > control.

The addition of a small amount of hydrocolloids resulted in a large enhancement in water-binding capacity and swelling capacity of starch pastes, but a reduction of syneresis in starch gels, indicating that the magnitude of such changes in paste rheology and texture depends on the type of starch and gum used [40,41]. The current results (PG group with highest water-binding capacity and swelling capacity) could be explained by a previous study which showed that the most pronounced effect has been the retardation of gelation kinetics of waxy maize starch plus the guar gum, rather than the combination with other gums [42].

Gelatinized waxy maize starch was subjected to different time and temperature conditions of storage to obtain samples with different extents of amylopectin retrogradation. Increased retrogradation extents reduced the enzyme susceptibilities to pancreatic alpha amylase and amyloglucosidase at 37 °C [43]. The pregelatinized starch produced more satiety than the raw potato starch, probably because this starch was digested too slowly to increase the postprandial blood glucose concentrations to any appreciable extent [44,45].

Soluble fiber with high water-binding capacity and viscosity may slow gastric emptying and small bowel transit [46,47], and several of the aforementioned signals generated in the gastrointestinal tract cooperate to promote satiety by activating vagal afferents and/or by acting directly on the brain [48,49]. Thus, the increased satiety by dietary supplementation of KF and PG is associated with decreased food intake in rats, which may be attributed to the high water-binding capacity and swelling capacity in those diets.

In the current study, supplementation of KF and PG significantly increased the concentrations of acetic acid, propionate acid, and SCFAs in the cecal content of rats. A number of factors—including chemical structure, particle size, surface area, and solubility of the fiber—affect the fermentation in the gut and the nature of the SCFAs produced [50,51]. Carbohydrates containing glucomannan or alpha-galacturonic acid residues are generally more susceptible to fermentation [51]. A comparison of *konjac* gum, guar gum, xanthan gum, and pectin in human fecal inoculum in a 48-h static fermentation culture system showed that all the gums increased the SCFAs level, and *konjac* and guar gum had the strongest acid-reducing ability among gum dietary fibers [52]. Resistant starch is completely degraded in the large bowel, and thus long-term consumption of fermentable fiber could produce satiety benefits [53].

It has been suggested that SCFAs stimulate the production and secretion of satiety-related hormones, such as GLP-1 and PYY, possibly via G-protein coupled receptors [54]. However, in the current study, dietary treatments did not alter the GLP-1 and PYY either in plasma concentration or mRNA abundance in distal ileum two hours after feeding, which is not consistent with a previous report that dietary supplementation of fructooligosaccharide increased the plasma concentration of PYY in the fasted state of rats [37]. It can be inferred that dietary fiber effect on satiety hormones (GLP-1/PYY) is associated with fed or fasted state. PYY and GLP-1 are both synthesized and released from L-cells located in the small intestine and large intestine [55]. They showed no increased expression here in distal ileum, probably due to an overall increase in L-cell number along the enlarged intestines, which was not detectable here since the real time PCR method measures expression per mg tissue. Slowly digestible starch inclusion in pig diet decreased distal ileum SCFAs transporter mRNA abundance [56]. This is why we selected the distal ileum for anatomical analysis.

The SCFAs produced via bacterial fermentation directly suppresses appetite via central hypothalamic mechanisms in rodents [57]. Recent findings from human study suggest that propionate production may play an important role in attenuating reward-based eating behavior via striatal pathways, independent of changes in plasma PYY and GLP-1 [58]. It is well known that SCFAs serve as an additional energy source, especially at the moment when glucose absorption is decreasing (or completed) in the small intestine, which helps to stabilize glucose levels in blood [59,60]. Delayed postprandial glucose transient declines could delay the onset of the ensuing meal and induce inter-meal satiety [61].

In the current study, dietary treatments had no effect on final body weight and body weight gain in the rats. At 10 weeks of age, the tested rats were young adults. The adult rats might continue to gain body mass at a slower rate, but the juvenile rapid growth phase ceased [62]. Therefore, the similarity in the body weight gain of rats among all the dietary treatments can be explained from the analysis of short-term effects.

The slight but not significant variation in feeding behavior, meal number, and SCFAs yield in PX group may result in an overall reduction trend but not in notable changes in food intake, probably due to the reason that the PX diet did not reach the level of KF and PG diets in the physicochemical properties (water-binding capacity and swelling capacity) and fermentability (SCFAs), thus it could not produce a significant effect on the satiety and food intake in rats.

In the current study, the daily dosage of soluble fiber per kilogram of body weight in rats was 1.4 g/kg (35 g (daily food intake) × 2% (dosage)/0.5 kg (body weight) = 1.4 g/kg body weight). Based on the dose translation formula (Human equivalent doses (mg/kg) = Animal dose (mg/kg) × (Animal *Km*/Human *Km*)) from animal to human studies revisited [63,64], the corresponding soluble fiber dose for humans was estimated to be 0.23 g/kg (1.4 g/kg (Animal dose) × 6 (Animal *Km*)/37 (Human *Km*) = 0.23 g/kg), which means the soluble fiber dose for a 60 kg person is 13.8 g (0.23 g/kg × 60 kg = 13.8 g). Further work is required to translate the findings to humans.

## 5. Conclusions

KF and PG reduce the food intake, probably by promoting the satiety, which was supported by decreased meal numbers and increased inter-meal intervals, but without a reduction in body weight gain. Additionally, dietary soluble fibers with high levels of physicochemical properties (water-binding capacity and swelling capacity) and fermentability (SCFAs) have a more significant effect on food intake, meal pattern, and feeding behavior in rats.

## Figures and Tables

**Table 1:** Ingredient and composition of experimental diets.

Ingredient (% *w*/*w*)	Control ^1^	KF ^1^	PG ^1^	PX ^1^
Corn	52.2	52.2	52.2	52.2
Soybean meal	15.9	15.9	15.9	15.9
Fish meal	10.0	10.0	10.0	10.0
Wheat bran	12.0	10.0	10.0	10.0
Sucrose	5.0	5.0	5.0	5.0
Fiber source		2.0	2.0	2.0
AIN-93 Mineral mix ^2^	3.5	3.5	3.5	3.5
AIN-93 Vitamin mix ^3^	1.0	1.0	1.0	1.0
**Composition**				
Crude protein (%)	20.04	19.94	19.78	19.90
Energy (kcal/g)	3.81	3.83	3.76	3.74
Soluble fiber (%)	1.91	2.89	3.09	2.57
Insoluble fiber (%)	10.46	10.58	10.61	11.10
Viscosity (mPa/s)	1.53	1.66	1.60	1.57
Swelling (mL/g)	1.81	2.63	3.03	2.18
Water-binding capacity (g/g)	2.05	2.58	2.87	2.26

^1^ Diets were control or supplemented with 2% fiber of *konjac* flour (KF), 2% pregelatinized waxy maize starch plus guar gum (PG) or 2% pregelatinized waxy maize starch plus xanthan gum (PX); ^2^ AIN-93 Mineral mix according to Reeves (1997), per kg mix: Calcium carbonate, 357.00; Potassium phosphate, 196.00; Potassium citrate, 70.78; Sodium chloride, 74.00; Potassium sulfate, 46.60; Magnesium oxide, 24.00; Ferric citrate, 6.06; Zinc carbonate, 1.65; Sodium meta-silicate, 1.45; Manganous carbonate, 0.63; Cupric carbonate, 0.30; Chromium potassium sulfate, 0.28; Boric acid, 0.08; Sodium fluoride, 0.06; Nickel carbonate, 0.03; Lithium chloride, 0.02; Sodium selenate, 0.01; Potassium iodate, 0.01; Ammonium paramolybdate, 0.008; Ammonium vanadate, 0.007; Powdered sucrose, 221.03; ^3^ AIN-93 Vitamin mix according to Reeves (1997). Vitamin (mg/kg) (except as noted): Nicotinic acid, 3.00; Ca pantothenate, 1.60; Pyridoxine, 0.70; Thiamin, 0.60; Folic acid, 0.20; Biotin, 0.02; Vitamin B_12_, 2.50; Vitamin E (500 IU/g), Vitamin A (500,000 IU/g), 0.80; Vitamin D_3_ (400,000 IU/g), 0.25; Vitamin K, 0.08; Powdered sucrose, 974.65.

**Table 2:** Behavioral satiety sequence of rats given diets containing different dietary fibers on day 15 ^1^.

Item	Control ^2^	KF ^2^	PG ^2^	PX ^2^	*p*-Value
Behavior (% time)					
Feeding	44.69 ± 3.45 ^a^	34.53 ± 3.06 ^b^	30.83 ± 3.19 ^b^	35.94 ± 2.94 ^a,b^	0.03
Drinking	6.04 ± 0.69	6.88 ± 0.70	5.47 ± 0.48	5.36 ± 0.80	0.38
Activity	21.15 ± 2.75	24.11 ± 3.54	21.82 ± 3.99	20.78 ± 2.12	0.88
Inactivity	10.42 ± 0.87	8.85 ± 1.52	10.73 ± 1.30	11.51 ± 2.01	0.64
Grooming	3.49 ± 0.47	4.53 ± 0.91	4.32 ± 1.09	4.11 ± 0.31	0.79
Resting	14.22 ± 4.08	21.09 ± 4.06	26.82 ± 4.28	22.29 ± 5.28	0.21

^1^ After a 12-h fast, rats were given food, and their behaviors monitored for 1 h. The percentage of time spent in each behavior was calculated and represented as mean ± SEM of *n* = 8 per group. Rows with different superscript letters indicate a significant difference (*p* < 0.05); ^2^ Diets were control or supplemented with 2% fiber of *konjac* flour (KF), pregelatinized waxy maize starch plus guar gum (PG), or pregelatinized waxy maize starch plus xanthan gum (PX).

**Table 3:** Meal pattern parameters of rats given diets containing different dietary fibers for three weeks ^1^.

Item	Control ^2^	KF ^2^	PG ^2^	PX ^2^	*p*-Value
Food intake (g/day)					
Total	51.68 ± 1.50 ^a^	46.58 ± 0.86 ^b^	46.01 ± 1.07 ^b^	48.47 ± 0.79 ^b^	<0.01
Nocturnal	23.40 ± 1.20	20.55 ± 1.04	20.79 ± 0.96	20.18 ± 1.53	0.24
Diurnal	28.29 ± 0.67	26.03 ± 1.33	25.22 ± 0.64	28.29 ± 1.62	0.16
Feeding rate (mg/s)					
Total	6.59 ± 0.93	6.54 ± 0.68	8.41 ± 1.29	5.89 ± 0.57	0.26
Nocturnal	5.67 ± 0.88	6.04 ± 0.44	7.09 ± 0.52	5.37 ± 0.62	0.26
Diurnal	6.59 ± 1.08	7.38 ± 1.25	8.47 ± 0.85	6.32 ± 0.60	0.45
Meal size (g/day)					
Total	3.12 ± 0.20	3.21 ± 0.13	3.39 ± 0.19	2.92 ± 0.19	0.33
Nocturnal	2.36 ± 0.14	2.53 ± 0.16	2.52 ± 0.15	2.20 ± 0.10	0.30
Diurnal	4.36 ± 0.41	4.22 ± 0.33	4.89 ± 0.47	3.73 ± 0.30	0.22
Meal duration (s)					
Total	507.45 ± 39.86	517.79 ± 41.60	437.35 ± 35.27	517.51 ± 41.47	0.43
Nocturnal	449.70 ± 38.00	440.70 ± 49.95	365.73 ± 28.00	437.96 ± 37.82	0.35
Diurnal	584.90 ± 49.69	620.19 ± 51.21	591.17 ± 40.64	612.64 ± 49.50	0.94
Meal number (meals/day)					
Total	16.88 ± 0.74 ^a^	14.63 ± 0.50 ^b^	13.88 ± 0.83 ^b^	17.00 ± 0.89 ^a^	0.01
Nocturnal	10.00 ± 0.38 ^a^	8.25 ± 0.41 ^b^	8.38 ± 0.50 ^b^	9.25 ± 0.70 ^a,b^	0.07
Diurnal	6.88 ± 0.61a ^b^	6.38 ± 0.50 ^a,b^	5.50 ± 0.53 ^b^	7.75 ± 0.41 ^a^	0.03
Inter-meal interval (min)					
Total	73.49 ± 4.57 ^b^	87.72 ± 4.16 ^a,b^	98.23 ± 9.48 ^a^	76.53 ± 4.03 ^b^	0.03
Nocturnal	58.34 ± 4.70	73.24 ± 6.40	70.90 ± 6.68	65.10 ± 4.74	0.27
Diurnal	106.65 ± 13.23	113.15 ± 9.48	133.19 ± 13.33	93.65 ± 5.61	0.09

^1^ Values are mean ± SEM of *n* = 8, rows with different superscript letters indicate a significant difference (*p* < 0.05); ^2^ Diets were control or supplemented with 2% fiber of *konjac* flour (KF), pregelatinized waxy maize starch plus guar gum (PG), or pregelatinized waxy maize starch plus xanthan gum (PX).
